# Interferon Gamma in Leishmaniasis

**DOI:** 10.3389/fimmu.2013.00156

**Published:** 2013-06-19

**Authors:** Peter E. Kima, Lynn Soong

**Affiliations:** ^1^Department of Microbiology and Cell Science, University of Florida, Gainesville, FL, USA; ^2^Department of Microbiology and Immunology, University of Texas Medical Branch, Galveston, TX, USA; ^3^Department of Pathology, University of Texas Medical Branch, Galveston, TX, USA

**Keywords:** leishmania immunology, cytokines and leishmania, chemokines and leishmania, signal transduction in infection, interferon gamma and leishmania

## Abstract

Leishmaniasis is a complex disease that is caused by parasites of the *Leishmania* genus. *Leishmania* are further classified into several complexes, each of which can engage in distinct interactions with mammalian hosts resulting in differing disease presentations. It is therefore not unexpected that host immune responses to *Leishmania* are variable. The induction of interferon gamma (IFN-γ) and response to it in these infections has received considerable attention. In this review, we summarize our current understanding of some of the host responses during *Leishmania* infections that are regulated by IFN-γ. In addition, studies that explore the nature of parasite-derived molecular mediators that might affect the host response to IFN-γ are also discussed.

## Introduction

Seminal studies on experimental infections of *Leishmania major* in C57BL/6 and BALB/c inbred mouse strains correlated parasite control with the elaboration of interferon gamma (IFN-γ) by CD4^+^ T cells and uncontrolled infections with the absence of IFN-γ in the C57BL/6 and BALB/c mice, respectively (Scott et al., 1989; Reiner and Locksley, 1995). Those historic studies of the immune response to *Leishmania* defined a disease model that supported the *in vitro* observations of Mosmann et al. (1986) that T cell responses can be subdivided on the basis of their cytokine production profile after re-stimulation. Attempts to explain the molecular basis of cytokine responses in *Leishmania* infections, their universality and the importance and potential for exploitation in efforts to control *Leishmania* infections has fueled numerous studies by immunologist; from an academic viewpoint, it has been that gift that has kept on giving. Outstanding questions that continue to be addressed are: (1) What factors limit the function of IFN-γ in the course of an infection? (2) When and how and does IFN-γ contribute to parasite control? (3) What mechanisms do parasites deploy to modulate either the environment in which IFN-γ has to function or modulate specific actions of IFN-γ? In this review, we summarize recent studies that have considered some of these questions. For a more complete assessment of the role of IFN-γ in leishmaniasis, other recent reviews on the immune response in *Leishmania* infections should be considered as well. Details on the biology of IFN-γ are presented in other contributions in this issue.

## Infections with *Leishmania* Species are Differentially Responsive to IFN-γ

There is ample evidence that IFN-γ and other cytokines that are secreted in the course of an infection have counter regulatory effects on the production and functions of each other. These counter-regulation mechanisms are in action at both the transcriptional and translational levels during T cell priming and differentiation stages for mouse and human CD4^+^ T helper (Th) subsets (Kaiko et al., 2008; Rautajoki et al., 2008). It is known that IFN-γ^−*/*−^ or IFN-γR^−*/*−^ C57BL/6 mice become highly susceptible to *L. major* infection, accompanied by an expansion of Th2-type responses (Swihart et al., 1995; Pinheiro and Rossi-Bergmann, 2007). However, IFN-γ^−*/*−^ C57BL/6 mice are as susceptible to *L. amazonensis* infection as the wild-type mice, at least for the first 2 months of infection, but they have more severe disease afterward (in the absence of an IL-4 dominance) (Pinheiro and Rossi-Bergmann, 2007). These observations indicate that IFN-γ is required for the control of *L. major* infection and can restrain pathogenic Th2 cell expansion, while IFN-γ is not essential for the control of *L. amazonensis* at the first few weeks of infection (Pinheiro and Rossi-Bergmann, 2007). These studies of *L. major* and *L. amazonensis* with side-by-side immunological studies in primary (Ji et al., 2003) and secondary infections (González-Lombana et al., 2008), suggest distinct mechanisms underlying host susceptibility to New World and Old World cutaneous leishmaniasis.

## Regulatory Role of IFN-γ in *Leishmania* Infections

Interferon gamma is not secreted in a vacuum; IL-10 and other Th2 cytokines exert critical effects on IFN-γ both at the level of induction and at the level of effector function, which then determine the course of infections. The reader is directed to recent reviews that have considered these interactions (Nylén and Sacks, 2007; Alexander and Brombacher, 2012). In this review, we will highlight recent findings regarding the cross-talk between IFN-γ and CXCR3-cognate chemokines and its impact on *Leishmania* infection. At present, much less information is known as to how IFN-γ regulates other pro-inflammatory chemokines in parasitic diseases. CXCR3 is a common primary receptor for three chemokines: CXCL9/Mig (also known as monokine induced by interferon gamma), CXCL10/IP-10 (also known as interferon-inducible protein-10), and CXCL11/I-TAC (also known as interferon-inducible T cell alpha-chemoattractant), which are structurally and functionally related molecules within the non-ELR CXC chemokine subgroup (Müller et al., 2010; Liu et al., 2011a,b). These chemokines are not constitutively expressed, but are up-regulated in a pro-inflammatory cytokine milieu, with a major function of selectively recruiting immune cells to inflammation sites. In the context of *Leishmania* infection, we and others have provided several lines of evidence that reveals a beneficial role of these CXC chemokines in parasite control.

The first line of evidence for a protective role of CXCL chemokines comes from *L. donovani* infection in mice. Earlier studies with IFN-γ^−*/*−^ mice and B6.Jalpha281^−*/*−^ mice (lacking NKT cells) have confirmed an absolute requirement for IFN-γ for sustained (24 h) expression of CXCL10 mRNA in *L. donovani* infection; an indispensable role for invariant NKT cells for the regulation of hepatic CXCL10 gene expression during the infection, because both CD3^−^NK1.1^+^ NK cells and CD3^*int*^NK1.1^+^ NKT cells are the early sources of IFN-γ production (Svensson et al., 2005). Recently, Gupta et al. (2011) have observed that CXCL10-treated, *L. donovani-*infected BALB/c mice displayed a strong host-protective Th1 response, accompanied by a marked decrease in immunosuppressive T_reg_ cells and TGF-β- and IL-10-secreting CD4^+^ T cells. Using a different approach, Majumder et al. (2012) have documented that CXCL10 is critical for the generation of protective CD8^+^ T cell responses induced by leishmanial antigen-pulsed, CpG-activated DC, because depletion of CXCL10 hampered CD8^+^ T cell activation and the generation of perforin and granzyme B. These studies suggest a role of CXCL chemokines on the regulatory functions of T cells and DC, which might be helpful in combating *Leishmania*-induced pathogenesis and boosting protective immunity. In contrast, it has been found that CXCR3^−*/*−^ mice can mount an efficient Th1 response, recruit T cells to the liver, and control *L. donovani* growth, even though they have a delayed onset of hepatic inflammation and granuloma formation (Barbi et al., 2007). The self-limiting nature of *L. donovani* infection in inbred strains of mice may partially account for the trivial protective effects in these knockout mice.

The second line of evidence for a protective role of CXCL10 comes from a murine model of *L. amazonensis* infection, for which nearly all inbred strains of mice are genetically susceptible to. We have revealed that pre-treatment with recombinant CXCL10 (100 ng/ml) significantly reduces *L. amazonensis* infection in bone marrow macrophages derived from BALB/c, C57BL/6, and C3H/HeJ mice, partially via a NO-mediated mechanism (Vasquez and Soong, 2006). Also, treatment with CXCL10 preferentially stimulates IL-12 production over IL-10 production in naïve and *L. amazonensis*-infected CD11c^+^CD45RB^−^ DC, and promotes IFN-γ production and IL-12Rβ2 chain expression by CD4^+^ T cells isolated from naïve or infected mice (Vasquez et al., 2008). The *in vitro* observations indicate positive feed-backs between IFN-γ and CXCL10 production for better control of the infection. Indeed, we have found that mice receiving subcutaneous injections of CXCL10 (100 ng) at days 1, 3, and 7 of infection with *L. amazonensis* have attenuated cutaneous lesions, as well as a 7- and 3.5-fold increase in IFN-γ and IL-12 production in re-stimulated lymph node cells (Vasquez and Soong, 2006). However, the major cellular sources of CXCL10 production during *Leishmania* infection *in vivo* have not yet been evaluated, although the top candidates may include CD4^+^ Th1 cells, CD8^+^ T cells, NK cells (Müller et al., 2010), NK T cells (Svensson et al., 2005; Beattie et al., 2010), as well as macrophages exposed to long-term culture (less-virulent) *Leishmania* (de Souza et al., 2010).

In agreement with the findings from murine infection models, clinical studies from healthy volunteers and leishmaniasis patients also suggest an involvement of CXCR3 chemokines in protection and disease pathogenesis. Several comparative studies have been conducted with *L. braziliensis* (the etiologic agent for cutaneous leishmaniasis and hyper-reactive mucosal leishmaniasis in South America), as well as *L. amazonensis* (the etiologic agent for cutaneous leishmaniasis and hypo-reactive disuse cutaneous leishmaniasis in South America). It has been reported that the crude antigen extracts of *L. braziliensis* (LbAg) are more potent than antigens of *L. amazonensis* (LaAg) in stimulating IFN-γ-producing cells and multi-functional CD4^+^ T cells of American cutaneous leishmaniasis patients (Macedo et al., 2012), and that LbAg also stimulate CXCL10 production in human PMBC, in a manner that correlates to the IFN-γ-positivity but not DTH-positivity (Schnorr et al., 2012). Using healthy donor PBMCs in parasite infection, we detected an enhanced expression of CXCL10 and other CC chemokines (CCL2-4) in responses to promastigote forms of *L. braziliensis* but not those of *L. amazonensis* (Vargas-Inchaustegui et al., 2010). Of note, CD14^+^ monocytes, but not CD3^+^ T cells or CD3^−^CD56^+^ NK cells, were the dominant cells that expressed CXCR3 on their cell surface and produced CXCL10 and IFN-γ in response to *L. braziliensis* infection *in vitro*. Given that serum samples from American tegumentary leishmaniasis (ATL) patients, especially in those of the mucosal leishmaniasis cases, have elevated levels of CXCL10, CCL4, and soluble tumor necrosis factor (TNF) receptor II (sTNFRII) (Vargas-Inchaustegui et al., 2010), and that the levels of CXCL9 were elevated in sera from *L. braziliensis-*infected, disseminated leishmaniasis patients, the biological functions of different CXCR3 chemokines during *Leishmania* infection in humans need to be carefully evaluated.

## Unresponsiveness of Infected Cells to IFN-γ

Cells infected with either the promastigote or amastigote forms of the parasite are less responsive to stimuli such as LPS that elicits IFN-γ production or IFN-γ itself that activates the anti-parasitic effector response of macrophages (Abu-Dayyeh et al., 2010; Matte and Descoteaux, 2010). Studies with mice that have a selective impairment of IFN-γ signaling in macrophage lineage cells have clearly demonstrated the critical role of IFN-γ-activated macrophages for the control of protozoan parasitic infections *in vivo* (Lykens et al., 2010). Studies assessing these responses have employed varying readouts to assess cell activation. These readouts have included evidence of transcription of expected genes, production of iNOS, evidence of translocation of signaling intermediates into the nucleus and phosphorylation of signaling intermediates in the cytosol. Each of these readouts has confirmed that *Leishmania* infection has a suppressive effect on responsiveness to IFN-γ. How and whether this reduced responsive of infected cells affects the course of infection is considered later. It is of interest that infections with amastigote and promastigote forms engage different mechanisms to accomplish the effect on the host cell. A study by Abu-Dayyeh et al., 2010 compared the effect of promastigotes or amastigotes on the activation of host protein tyrosine phosphatase (PTP), activation of the transcription factors: signal transducers and activator of transcription (STAT); AP-1 and NF-κB, which are signaling intermediates that have been shown to mediate the effects of LPS and IFN-γ (Zhang and Ghosh, 2000; Johnson et al., 2012). In agreement with previous studies, they observed that both parasite forms inhibit the activation of STAT1 and AP-1. Furthermore, infection with both parasite forms blocked NF-κB translocation into the nucleus by degradation of the p65 subunit; interestingly, in light of the fact that the products of p65 degradation was different depending the infecting parasite form, it was proposed that distinct parasite molecules might be responsible for targeting NF-κB activation. Our knowledge of the identity of such molecules is still limited. The only molecule that has been characterized with this effector function is the cysteine proteinase (LmCPb). Abu-Dayyeh et al., showed that this molecule not only targets NF-κB but it also targets PTP-1b. How LmCPb recognizes these targets is presently not known. Moreover, the mechanism by which this and other parasite-derived molecules that are expressed within the parasitophorous vacuole access targets in the cytosol has thus far been a matter of speculation (Mottram et al., 2004). In a different study that evaluated the mechanism by which amastigote forms limit the responsiveness of infected cells, the function of importin-α5 that mediates the translocation of STAT1 among other transcription factors from the cytosol to the nucleus was shown to be inhibited, which prevented the activation of Interferon regulatory factor 1 (IRF-1) (Matte and Descoteaux, 2010). The authors speculated on the likely existence of parasite-derived molecules that target importin-α5 function.

In light of the evidence that *Leishmania* infection reduces responsiveness of infected cells to IFN-γ, it is not known what role the remnant muted response plays in the development of a *Leishmania* lesion. Since responses to cytokines are dependent on the concentration of the cytokine, the local concentration of IFN-γ might determine in large part what will be the cellular responses there. Obtaining a greater understanding of the microanatomy of IFN-γ and other CD4^+^ T cell effector activity at the site of a lesion is therefore of great interest. Delivery of cytokines such as IFN-γ is known to be strictly limited to the immunological synapse *in vitro* (Huse et al., 2006). By using static imaging of effector CD4^+^ T cells in bacille calmette–Guérin (BC2013G)-induced granulomas, Egen et al. (2011) have shown that IFN-γ is localized at the T cell-antigen-presenting cell (APC) interface *in vivo*, suggesting a strictly localized effector activity at the site of infection. However, this view of “high local specificity for delivered cytokines” is challenged and greatly expanded by a recent study of Müller et al., that involves a self-healing mouse model of cutaneous *L. major* infection. In this study, the authors asked whether CD4^+^ T cells have to engage each *L. major*-infected cell individually in order for efficient parasite clearance (Müller et al., 2012). They found that although CD4^+^ T cells formed stable contacts with a limited number (10%) of infected cells, such contacts efficiently activated intracellular defense mechanisms in infected cells by producing a gradient of effector signals (including IFN-γ and iNOS expression), which would extend more than 80 μm beyond the site of T cell-APC interactions. This implied that infected cells within that range can respond to those IFN-γ levels to elaborate anti-*Leishmania* mechanisms. These findings are exciting as they help us understand some issues that were raised by previous observations. Specifically, several reports had shown that infected cells harboring live parasites are poor antigen presenters of endogenously synthesized parasite antigens to CD4^+^ T cells (Kima et al., 1996; Prina et al., 1996), which implied that cytokine production within the lesion would be limited. With the results of Müller et al., it is possible to envision a scenario in which APCs that acquire antigens from dead parasites can activate CD4^+^ T cells to unleash cytokines that limit the development of the lesion. The studies of Müller et al., also shed new light on immune-mediated parasite control in self-healing cutaneous leishmaniasis that are known to trigger strong and sustained IFN-γ production for efficient control of *L. major* and *L. braziliensis* infection in genetically resistant mouse strains (Pagan et al., 2013). In these self-healing mouse models, and possibly subclinical human infections, CD4^+^ T cells can extend their effector functions beyond the immunological synapse and activate bystander effector activities, including CXCL10and iNOS, to control intracellular pathogens.

This view of cytokine gradient-based parasite control also helps understand chronic and non-healing leishmaniasis associated with *L. donovani*, *L. amazonensis*, and *L. mexicana* infections in mouse and humans. Since these parasite species can persist for months or years and replicate in target cells in the face of appreciable levels of IFN-γ, we envision several possible mechanisms for non-healing infections. Firstly, the concentration of IFN-γ may not be sufficiently high, or the duration of IFN-γ production may not be sustained long enough, to reach other infected or bystander cells in distance. This possibility is supported by many *in vitro* studies at the single-cell level (Vargas-Inchaustegui et al., 2008, 2009; Pereira et al., 2009; Xin et al., 2011; Macedo et al., 2012). Secondly, the expression of IFN-γ receptor may not be sufficiently high in bystander cells to respond to IFN-γ. The depressed expression of IFN-γ receptor in infected cells have been reported (Ji et al., 2003), but evidence for IFN-γ receptor expression in bystander cells, or for local unresponsiveness, is still missing. Further evaluation at the tissue and molecular levels, either via immuno-histochemical staining or *in situ* RT-PCR analysis, will be helpful to test this possibility. Thirdly, IFN-γ production by itself is not sufficient to activate the effector functions in infected or nearly bystander cells. An increase in IFN-γ single-positive T cells by itself is not sufficient to protect mice against primary infection, or predict vaccine-based protection in humans and in mice (Xin et al., 2011; Macedo et al., 2012). Efficient control of these parasite species, therefore, needs multiple pro-inflammatory cytokines (IFN-γ, IL-2, and TNF-α), as well as leishmaniacidal chemokines such as CXCL10 (de Souza et al., 2010; Vargas-Inchaustegui et al., 2010). Finally, there is increasing evidence that some parasite species/stages (such as amastigotes of *L. amazonensis*) are relatively resistant to IFN-γ- and NO/ROS-mediated killing in infected macrophages (Qi et al., 2004; Wanasen et al., 2007), as well as in cell-free medium (Soong et al., 2012). While much higher levels of effector molecules are required to kill parasites in the *L. donovani* and *L. mexicana* complexes, these parasites are often highly competent in repressing host Th1-type responses or utilizing such responses for immunopathogenesis (Soong et al., 2012).

## Conclusion

Interferon gamma plays important roles in the development and subsequent control of *Leishmania* infections. It and other cytokines and chemokines exert effects on each other that influence the course of infection. *Leishmania* parasites elaborate factors that modulate activities induced by IFN-γ. It is anticipated that greater insight into these interactions will be obtained in future studies of *Leishmania* lesions at the cellular level.

## Conflict of Interest Statement

The authors declare that the research was conducted in the absence of any commercial or financial relationships that could be construed as a potential conflict of interest.
